# A Real Man!

**DOI:** 10.3402/gha.v9.34140

**Published:** 2016-12-14

**Authors:** Aswini Kumar Behera, Arima Mishra, Sreeparna Chattopadhyay

**Affiliations:** Coordinator, Health and Nutrition Project Gram Vikas, Bhubaneshwar, Odisha, India, Email: aswini.behera14@apu.edu.in; Faculty, Azim Premji University, Bangalore, India, Email: arima.mishra@apu.edu.in; Azim Premji University, Bangalore, India, Email: sreeparna.chattopadhyay@apu.edu.in

**Figure:**
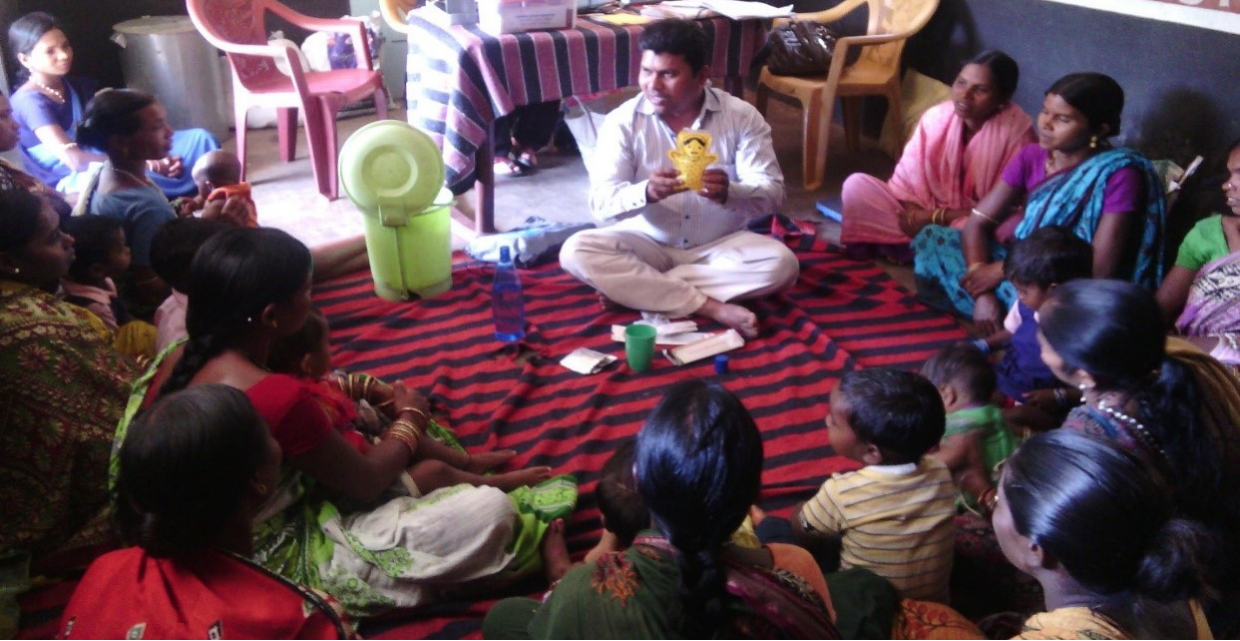


Arjun* is a community health worker who has been working in a remote, hilly region in the state of Odisha, India, for the past 10 years. This region is primarily inhabited by indigenous communities, known as Adivasis, and has had relatively poorer health outcomes until the past decade, when the Government of India introduced several health system reforms to strengthen public health service delivery.

Arjun is addressing the tribal women during the ‘Village Health and Nutrition Day’ (a monthly outreach clinic) and demonstrating how to protect children from diarrhea, one of the most common diseases among children in the region and a leading cause of child mortality. Women are paying rapt attention as they listen to his messages. Arjun defies gender stereotypes through his sheer commitment to a profession which is otherwise dominated by women. His sincerity and dedication to the cause of improving the health of the community not only has earned the respect and trust of the village women but also the ‘Best Health Worker’ award from the government in recognition of his efforts.

The photograph was clicked by the first author as part of a study aiming to understand maternal and child health practices among an indigenous community in this region. The study involved, apart from interviewing the women, observations of outreach clinics and accompanying the health workers in their everyday work. Community health workers play an important role as an interface between the community and the health system. Women's access to routine maternal health services, including antenatal care, family planning, and immunization, has reasonably increased with the regular holding of the outreach clinics in the village, although accessing emergency obstetric services continues to be a concern. For many of these Adivasi women, the outreach clinics and interactions with health workers provide a forum for systematic engagement with biomedicine and the state. The study was conducted from September, 2015 to January 2016 and was jointly supervised by the second and third authors.

*Aswini Kumar Behera* Coordinator, Health and Nutrition Project Gram Vikas Bhubaneshwar, Odisha, India Email: aswini.behera14@apu.edu.in*Arima Mishra* Faculty, Azim Premji University Bangalore, India Email: arima.mishra@apu.edu.in*Sreeparna Chattopadhyay* Azim Premji University Bangalore, India Email: sreeparna.chattopadhyay@apu.edu.in

## Footnotes

* pseudonym

